# Tracing the Rise of Ants - Out of the Ground

**DOI:** 10.1371/journal.pone.0084012

**Published:** 2013-12-26

**Authors:** Andrea Lucky, Michelle D. Trautwein, Benoit S. Guénard, Michael D. Weiser, Robert R. Dunn

**Affiliations:** 1 Department of Biology, North Carolina State University, Raleigh, North Carolina, United States of America; 2 Entomology and Nematology Department, University of Florida, Gainesville, Florida, United States of America; 3 Department of Entomology, North Carolina State University, Raleigh, North Carolina, United States of America; 4 Nature Research Center, North Carolina Museum of Natural Sciences, Raleigh, North Carolina, United States of America; 5 Biodiversity and Biocomplexity Unit, Okinawa Institute of Science & Technology, Onna-son, Okinawa, Japan; 6 Department of Biology, University of Oklahoma, Norman, Oklahoma, United States of America; Field Museum of Natural History, United States of America

## Abstract

The evolution of ants (Hymenoptera: Formicidae) is increasingly well-understood due to recent phylogenetic analyses, along with estimates of divergence times and diversification rates. Yet, leading hypotheses regarding the ancestral habitat of ants conflict with new findings that early ant lineages are cryptic and subterranean. Where the ants evolved, in respect to habitat, and how habitat shifts took place over time have not been formally tested. Here, we reconstruct the habitat transitions of crown-group ants through time, focusing on where they nest and forage (in the canopy, litter, or soil). Based on ancestral character reconstructions, we show that in contrast to the current consensus based on verbal arguments that ants evolved in tropical leaf litter, the soil is supported as the ancestral stratum of all ants. We also find subsequent movements up into the litter and, in some cases, into the canopy. Given the global importance of ants, because of their diversity, ecological influence and status as the most successful eusocial lineage on Earth, understanding the early evolution of this lineage provides insight into the factors that made this group so successful today.

## Introduction

The precise habitats and conditions favoring the rise of one of the most successful groups of social organisms on earth, ants, have long been a subject of fascination and study [1], [2]. Despite the large body of research on this topic [3]–[6], it has been difficult to reconstruct the ancestral environment, and specifically the type of habitat in which the first societies of ants arose. Recently, however, the simultaneous growth in the quality and comprehensiveness of the phylogenetic trees and data on the distribution and life history of individual ant taxa allow us to test hypotheses about the conditions under which the ants evolved [5].

Today, ants occupy nearly every stratum of terrestrial ecosystems from the deep soil to the highest forest canopy, from the tropics to the subarctic and subantarctic [7] such that they often play a dominant role in ecosystems in terms of their diversity, abundance and ubiquity. This, however, was not always the case. Ants are a monophyletic group and so necessarily arose from a single lineage in a specific habitat or habitats. Crown group ants originated 139–158 million years ago (Mya) [8]–[12]. For most of their evolutionary history, they appear to have been relatively rare and attained their modern abundance only more recently [13], [14], perhaps as late as 50 Mya, in the mid-Eocene. At some point during these millions of years, ants made transitions out of their ancestral habitat stratum (or strata), whether that was belowground, above the soil surface or in the trees.

In the most prominent model of early ant evolution, Wilson and Hölldobler [4] offer a ‘Dynastic Succession’ hypothesis, which introduces the idea that ants arose in the leaf-litter and soil of tropical angiosperm forests and then spread to other strata (including into the soil) and other biomes. The biome aspect of this hypothesis was recently addressed in a paper by Moreau and Bell [15], who presented convincing evidence that the tropics (specifically the Neotropics) played an important role in the early evolution and diversification of ants. To date, however, there has been no explicit test of the habitat hypothesis, though it is often verbally considered, e.g. [5], [16], [17]. Recently, the discovery of both a new basal lineage of subterranean, predatory ants [16], and new fossil deposits [11], [18], [19] have re-ignited focus on the ecology of the earliest ant lineages. These new findings have prompted further discussion of the habitat strata in which early ants lived, including speculation about possible arboreality in some of the earliest ant fossils [20].

Here, we statistically assess the predictions of the Dynastic Succession hypothesis as they relate to habitat strata for the first time. We test the hypothesis that ants arose in the leaf litter, as well as the alternative ‘Out of the Ground’ hypothesis that ants evolved in the soil and then, secondarily, colonized the leaf-litter and other strata. To do this we reconstruct ancestral states of habitat strata on the ant tree of life, and examine historical transitions between states to reveal which are likely to be ancestral to all ants and which, if any, appear only more recently. We also consider the relative frequency (and arguably, ease) with which ant lineages have made transitions among different habitat strata.

## Methods

### Phylogeny-based character assignments

In order to reconstruct the ancestral traits of ants, we mapped characters onto terminal nodes of the phylogeny of Brady et al. [9]; similar topologies were recovered in [5], [16], [15]. Included are 151 species, representing 134 ant genera (out of 308) belonging to 20 of 21 extant ant subfamilies [21], (see Table S1 in File S1 for genera included here and habitat references). Multiple outgroups in Brady et al. were pruned from this tree so that only a single outgroup taxon remained (*Apis mellifera*) [22].

Each terminal was assigned a state for habitat strata and for biome that reflects the known biology for the genus based on literature records and consultations with experts [23]. The included genera represent approximately half of the known ant genera and are broadly representative of ant ecological diversity (Table 1): 35% of the genera examined here live and forage primarily on the soil surface stratum (above the mineral layer); fewer are exclusive to the soil's low-light environment (23.6%), and fewer still occupy forest canopies (14.6%). The remainder occupies multiple strata (soil+litter: 11.8%; litter+arboreal: 12.5%; all strata: 2.1%). These genera are also reflective of the geographic distribution of ant diversity (Table 1): The majority of ant genera span ranges that encompass Tropical, Subtropical and Temperate regions (59.7%); a smaller proportion are found in Tropical and Subtropical regions (35.4%); very few are limited to Subtropical and Temperate regions (3.5%) or solely Subtropical regions (1.4%); no ant genera occur exclusively in Temperate zones.

**Table 1:** Relative proportions of extant ant genera occupying each habitat stratum and biome category.

Strata (%)		Biome (%)		Any Soil spp. (%)		Any Arboreality (%)	
Soil only	23.6	Trop+Subtrop	35.4	Any Soil	39.6	Any Arboreal	30.6
Soil+Litter	11.8	Subtrop	1.4	No Soil	60.4	No Arboreal	69.4
Litter only	35.4	Subtrop+Temp	3.5				
Litter+Arboreal	12.5	Trop+Subtrop+Temp	59.7				
Arboreal only	14.6						
Soil+Litter+Arb	2.1						

Relative proportions of the 134 included ant genera occupying each habitat stratum and biome category. Highest proportions are in bold.

### Habitat Strata

All ant genera were coded as being soil dwelling, surface dwelling, arboreal or some combination of these three. We categorized ant genera according to the habitat strata in which they nest and forage, with an emphasis on light availability to distinguish soil-dwelling ants from those dwelling on the surface. The commonly used designation of ‘leaf litter dwelling’ does not clearly differentiate these differences, so we avoid using that terminology here. Instead, we define subterranean genera as those which nest and forage in a primarily low-light environment below the surface stratum. Surface dwelling genera forage on the surface of the soil or in the leaf litter, exposed to open air and direct sun- or moonlight; most of these genera nest underground or in protected spaces aboveground that are contiguous with the soil (e.g. rotting logs). Arboreal genera nest within living or dead tissue of standing trees.

We also used binary coding schemes to code taxa according to whether or not the lineage (genus) possessed any species that were soil dwelling as well as whether any arboreality was present in the lineage. Coding schemes used were 1) any soil-dwelling species vs. none and 2) any arboreal species vs. none.

### Biome

We examined biome at a coarse scale. Terminals were assigned one of four character states for biome: 1) tropical+subtropical, 2) subtropical, 3) subtropical+temperate, and 4) tropical+subtropical+temperate (no terminals were found to be solely tropical, solely temperate or tropical+temperate). Occurrence data were assessed at the level of political boundaries (primarily country) from the literature and expert opinion [24]. Latitudes from 0.0°–23.5° (North and South) were designated as Tropical; 23.5°–40.0° as Subtropical; >40.0° as Temperate. In assigning taxa to biomes, we used current biome as an approximation of the physiological tolerance of lineages [25]. With the exception of the very coldest environments present today, all modern biomes have at least coarse analogues during both the Cretaceous origin and early diversification of ants and Eocene rise in their abundance [26].

### Phylogenetic Uncertainty of Basal Ant Lineages

We evaluated the evolution of strata and biome preferences by mapping life history data onto trees that represent consensus views of ant phylogeny [5], [9], [16], [27]. Because evolutionary relationships among the earliest ant lineages remain controversial, a first and necessary step toward reconstructing the evolution of the ancestral habitat affinities of ants was to revisit the placement of these lineages. The analyses of Brady et al. [9] exhibited uncertainties regarding the placement of leptanillines as either the sister group to all ants or as a member of the poneroid clade. In light of this uncertainty, which can impact ancestral state reconstruction, we assessed the robustness of the placement of Leptanillinae using a maximum likelihood quartet-puzzling approach that allowed us to visualize the phylogenetic signal for conflicting hypotheses of Leptanilinae placement. We performed a four-cluster likelihood-mapping analysis in Tree Puzzle [28] using the original nucleotide data set of Brady et al. [9] to visualize affinity between 1) poneroids, 2) formicoids, 3) leptanillines, and 4) outgroups. If the placement of leptanillines as the sister group to all ants was a methodological artifact (i.e. long branch attraction), the likelihood mapping analysis would reveal conflicting signal for Leptanillinae placement, revealing affinity for both the outgroups as well as poneroids.

### Phylogenetic Signal

Phylogenetic signal of the characters mapped onto the phylogeny was calculated using Blomberg's K statistic [29], implemented in Picante [30] within the program R. Significance was based on 1000 reshuffled tip states, with an expectation based on tree structure and assuming Brownian motion character evolution. To ensure that our results were not biased by using Brownian motion to model discrete data, we also tested phylogenetic signal using Pagel's lamda statistic using the program Geiger in the R platform [31], [32]. To test for significance, we used a likelihood ratio test to compare the likelihood of the original tree with that of a lamda-transformed tree with no phylogenetic signal.

### Ancestral State Reconstructions (ASR)

**Figure 1:**
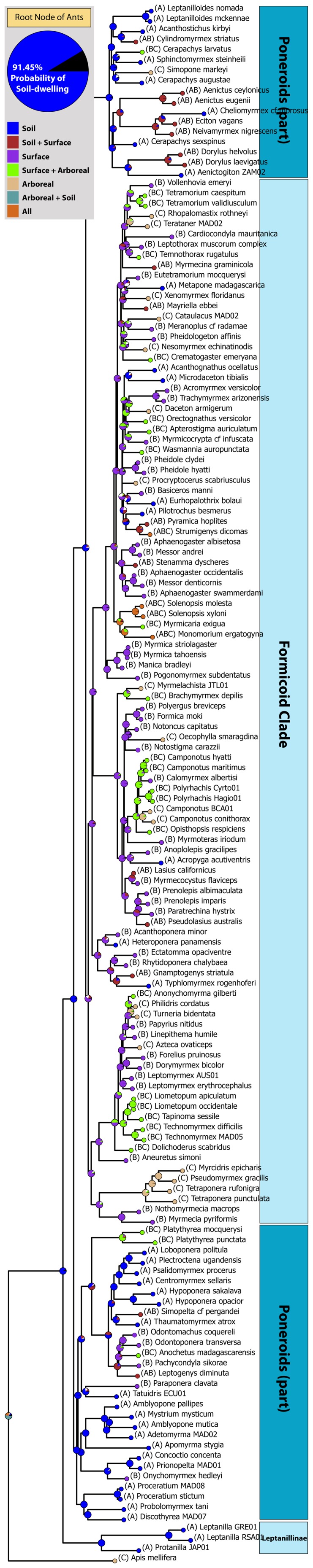
Ancestral state reconstruction of ant habitat. Ancestral state reconstruction of ant habitat strata based on phylogram plus 500 trees sampled from the original likelihood distribution of Brady et al. [9]. The outgroup in this reconstruction performed in the program RASP was coded as soil-dwelling, surface-dwelling or arboreal. The root node of ants is reconstructed as being subterranean (soil dwelling) with a posterior probability of 91.45%.

Ancestral character states were reconstructed for habitat strata using several different approaches in the programs RASP and BayesTraits. All analyses were based on topologies of 500 trees filtered from the posterior distribution of Bayesian analyses done by Brady et al. [9], with outgroups pruned to a single taxon (*Apis mellifera*). Outgroup pruning ensured trees were strictly bifurcating (a requirement of BayesTraits). Analysis of a sample of trees (in contrast to analyses based on a single consensus tree) takes into account phylogenetic uncertainty, varying branch lengths and transition rates. In order to account for the fact that ancestral state reconstructions treat each branch as a single terminal with polymorphic states, and acknowledge that our coding scheme may suggest greater intraspecific polymorphism than actually exists, we used the F81+G model in the program RASP that explicitly allows for multiple overlapping character states [33] to reconstruct ancestral character states for all internal nodes (Table 2, Fig 1). To confirm the strata for the most recent common ancestor of all ants, we performed additional Bayesian and Likelihood analyses in BayesTraits. For Bayesian analyses, Markov Chains Monte Carlo (MCMC) were run for 5 million iterations and sampled every 300^th^ run. Bayesian priors were chosen based on rate coefficient estimates from maximum likelihood runs. Additional analyses based on binary coding of characters (e.g. soil-dwelling vs. not soil-dwelling) were also completed.

**Table 2:** Influence of outgroup coding on Ancestral State Reconstruction.

Trait Reconstructed (Soil, Surface or Arboreal)	Outgroup coding (Soil, Surface or Arboreal)	Posterior Probability
Soil	Soil	0.99
Soil	Surface	0.86
Soil	Arboreal	0.70
Soil	Soil & Surface & Arboreal	0.92
Soil	Soil & Surface	0.97
Soil	Surface & Arboreal	0.79
Soil	Soil & Arboreal	0.91
Soil	Null (Neither soil, surface nor arboreal)	0.80

Influence of outgroup coding on Ancestral State Reconstructions of the habitat stratum of the root node of ants, with MCMC posterior probabilities provided as support values (performed using the Bayesian Binary Method in RASP using the model F81+G). In all cases soil was reconstructed as the ancestral habitat with the highest proportional likelihood (vs. surface dwelling or arboreal).

### Transitions

Changes of state were summarized over the phylogeny by assessing relative MCMC transition rate coefficients on all 500 trees (as described above) in BayesTraits. ‘Global’ transition rates between characters were optimized in analyses that did not include specific ancestral node reconstructions. Timing of colonizations of different habitat strata was established by calculating a single dated chronogram representing the mean of minimum and maximum trees [9]. Ancestral state reconstructions were performed on the phylogram, and subsequently correlated to these mean ages.

### Influence of Outgroup Coding

In order to address concerns how about outgroup coding affected our results, we performed ancestral state reconstruction in RASP with the outgroup coded to all possible ancestral states, using every possible combination of Soil, Surface and Arboreal as well as null (neither Soil, Surface nor Arboreal).

## Results and Discussion

The idea that that ants evolved as a clade adapted to the tropical leaf-litter and soil and subsequently made colonization events both out of the tropics and out of the litter into forest canopies and deep into the soil was presented by Wilson and Hölldobler [4] and later supported by findings of other authors, e.g. [5], [15], [16]. Yet it was never quantitatively tested until now. Our results refine the dynastic succession hypothesis, and suggest an ‘Out of the Ground’ progression in which ants arose in the soil and only later made transitions into the leaf-litter and other strata. Essentially, the story of the rise of ants is one that began in the early Cretaceous soil (139–158 Ma [5], [9]), with ants subsequently emerging on the surface stratum. Once on the surface, most of the diversification of the modern (crown group) genera took place, and many lineages subsequently transitioned among different strata and biomes.

### Revisiting the earliest ant divergences

**Figure 2:**
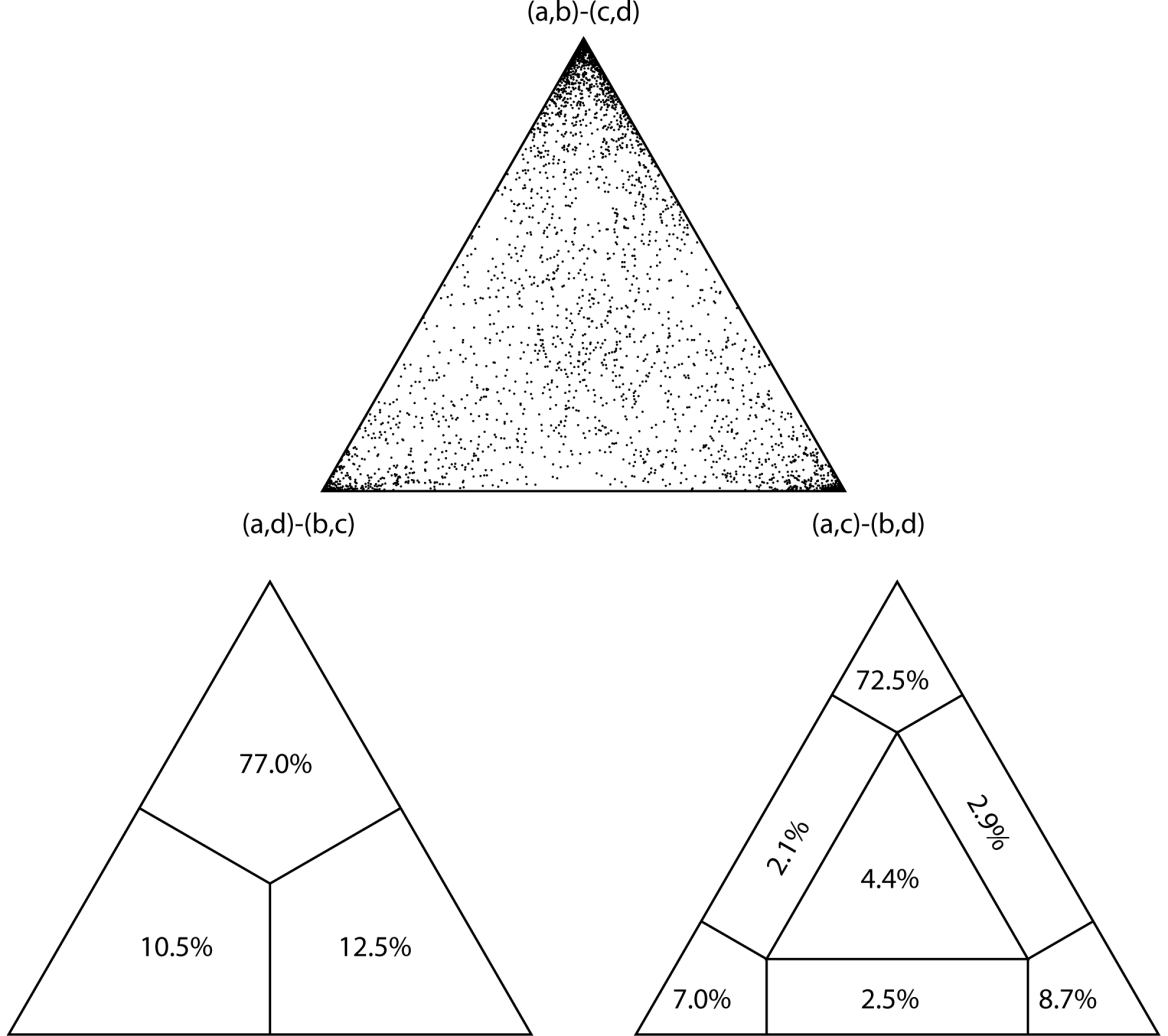
Support for Leptanillinae as sister to all other ants. Three depictions of a four-cluster likelihood map visualizing the strength of the phylogenetic signal supporting the placement of lepantillines outside the poneroid + formicoid clade. The top triangle indicates density of individual reconstructions; bottom left and right indicate percentage of points in divided cluster-space, with each tip representing a particular hypothesis (or specific arrangement of taxa). a: outgroups, b: leptanillines, c: poneroids, d: formicoids.

We revisited uncertainties raised about the relationships among the earliest ant lineages [9], [15], [16], [24] because of the important influence of tree topology on ancestral state reconstructions. We specifically addressed the question of whether the subfamily Leptanillinae was strongly supported as sister to all other ants. To visualize the strength of the phylogenetic signal supporting competing hypotheses for leptanilline placement, we performed a four-cluster likelihood-mapping analysis of the nucleotide dataset of Brady et al. [9] (Ants were clustered in four groups: Poneroids, Formicoids, Leptanillines and outgroups). Our results show strong affinity (80.7%) between the subterranean, tropical Leptanillinae and outgroups (Figure 2), and between the poneroid clade and the formicoid clade. These findings reveal no conflicting signal, which would be expected if long-branch attraction, the primary concern raised about this arrangement, were responsible for erroneously placing Leptanillinae as the sister group to all other ants. These results, along with the consistent recovery of similar phylogenies from other molecular studies [15], [16], together support Leptanillines as sister to the remaining ants, and provide additional assurance that the Brady et al. [9] phylogeny is a robust tree on which to test ecological and evolutionary hypotheses.

#### Ants come out of the ground

The reconstruction of the ancestral traits of early ants strongly suggests that the most likely habitat stratum for the ancestral ant was within the soil with subsequent, and perhaps multiple, colonizations of the surface stratum and, in some cases, forest canopies (Figure 1, Table 3; Posterior probability 0.9145). These results were also supported when we used simple binary character states for strata (under- vs. above-ground; proportional likelihood 0.6448), as opposed to six-character habitat states. Bayesian and ML reconstructions of the most recent common ancestor of all ants based on these 500 trees from the posterior distribution of the Bayesian analysis of Brady et al [9] support findings that the earliest ants likely lived a subterranean lifestyle (Table 3, Table S2 in File S1).

**Table 3:** Ancestral state reconstruction of the habitat stratum of the root node of ants.

Trait Reconstructed		MCMC Posterior probability	ML Mean proportional likelihood
*Strata* (6-state)	Soil	0.695	0.631
	Soil/Litter	0.125	0.100
	Litter only	0.053	0.069
	Litter/Arboreal	0.038	0.060
	Arboreal	0.031	0.057
	All	0.058	0.084
*Strata* (Binary)	Soil-dwelling		0.645
	Not soil-dwelling		0.355
	Arboreal		0.310
	Not Arboreal		0.690

Ancestral state reconstruction of the habitat stratum of the root node of ants. Support is given as MCMC posterior probabilities and ML mean proportional likelihoods (performed in BayesTraits).

We also assessed the influence of outgroup coding on our results in order to consider the possibility that reconstruction of the ancestral stratum of ants is contingent on the strata assigned to outgroups. We found that all possible outgroup coding schemes supported a subterranean habit for the earliest ants (79–99% probability for subterranean habits) (Table 2). Even when the outgroup was coded as being non-subterranean (e.g. arboreal and or surface-dwelling), we still found strong support for the earliest ant lineage being subterranean, underscoring the robustness of this result.

**Figure 3:**
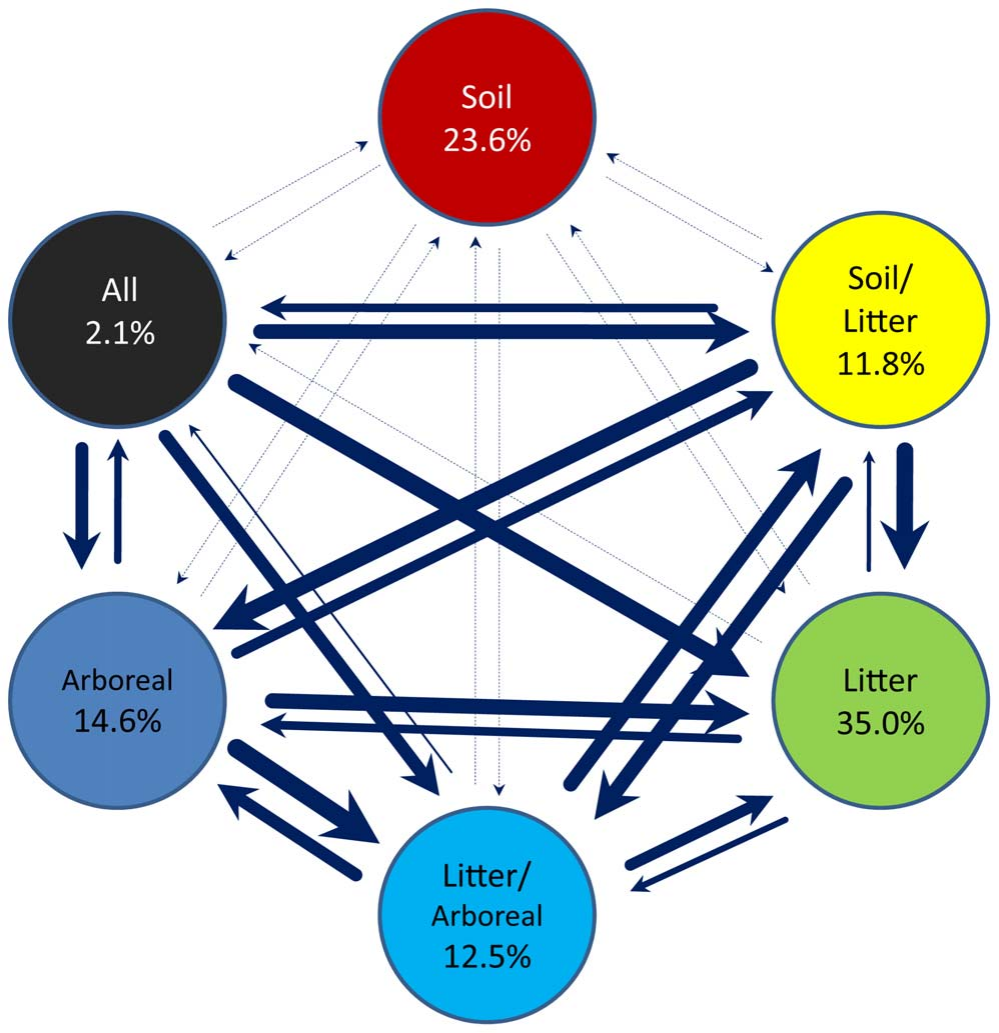
Evolutionary transitions among habitat strata. Rate of transitions among six habitat states summarized across 500 trees sampled from the original likelihood distribution of Brady et al. [9]. Thickness of arrows corresponds to rate of transitions. The percentages of ant genera that occupy a habitat category are indicated within each circle.

After ants emerged from the ground, many secondary transitions occurred, but some transitions were more likely than others. Figure 3 depicts relative frequency of transitions from one state to another, based on median Bayesian rate coefficient values from global BayesTraits analyses (Table S2 in File S1). In reconstructions of habitat strata the most frequent transitions were amongst above-ground states, with transitions to and from soil exclusively being extremely infrequent.

The relative rarity of transitions between strictly above- and below-ground habits on the ant tree of life suggests that such transitions are evolutionarily difficult, and likely require more physiological and behavioral changes than are required for transitions within either realm (from surface-dwelling to arboreal, for example). The leaf litter habitat that emerged with the prominence of angiosperm forests may have been influential in easing transitions between the surface strata and soil environment as it incorporated elements of both strata and offered a protected transition zone between the two.

Significant phylogenetic signal for habitat strata in both 6-state and binary coding schemes suggests that species dwelling and foraging below the soil surface are subject to stronger phylogenetic constraint than those that occupy above-ground realms, including the leaf litter and in the trees (6-state: K = 0.4336, p = 0.0001; soil-dwelling: K = 0.3631, p = 0.0001; arboreal: K = 0.1529, p = 0.0004). These results were further supported by assessment using Pagel's lambda statistic, which also showed strong phylogenetic signal (P = 8.5022E-18) for the 6-state habitat strata (⋌ = 0.9323). A likelihood ratio test indicated a significant difference in likelihood scores for the original tree with phylogenetic signal (lnl = −259.5393) as compared to the lamda transformed tree with no phylogenetic signal (lnl = −222.6229). Additionally, significant shifts in diversification rate reported in previous studies correlate with some important habitat shifts, such as the move to partial or complete arboreality within clades (e.g. the tribe Camponotini in Formicinae, the genus *Crematogaster*, the subfamily Dolichoderinae; [5], [9], [15]). The timing of diversification of crown group ants may correspond to habitat shifts in ants that were prompted by and led to increased dependence on trees and forested habitats [15].

#### A new evolutionary context for the origin of ants

The hypothesis that ants originated in tropical leaf litter [4] emerged prior to the reconstruction of a robust molecular phylogeny of ants. It was based on early attempts to date ant origins [3] as well as morphology of the oldest ant and stem-group ant fossils, about which much more is known today [34]–[38]. Recently, the age of the earliest ants and the subsequent diversification of the family were estimated with molecular and fossil evidence [5], [9], [15]. The earliest ants, fossils from the Cretaceous, are indeed dissimilar to the extant lineages known now to be sister to all the remaining ants [11], [18], [19].

The earliest fossils representing the presumed sister-group to ants, the extinct subfamily Sphecomyrminae, are known from workers and have large eyes, suggesting they were not soil-dwelling, but rather epigaeic or arboreal [39]. However, fossils of the earliest ant progenitors are scarce and might not be recognized as soil-dwelling. In particular, reproductives may not have displayed specialized traits (such as eyelessness, paleness) which characterize many modern subterranean ants [40]. Even today, reproductives of many subterranean species possess fully functional eyes and robust bodies (e.g. some *Cerapachys* spp.). Interpretation of the function of appendages, as in the bizarre trapjaw-like mandibles of the early fossil genus *Haidomyrmex* [20], highlights the difficulty of associating fossil species with habitat. These factors, combined with other hints about early ant lifestyles, make it tempting but difficult to draw clear conclusions about habitat use based on early ant fossils. For example, Poneromorphs are represented in amber mostly by alates [18], [36], [37], indicating that workers were soil dwelling, whereas the workers of surface-dwelling species (Formicinae and Dolichoderinae) are primarily preserved in the amber fossil record and thought to be arboreal [38]. Finally, the subterranean habitat is frequented by the descendants of the lineage from which early ants evolved, the aculeate Hymenoptera. Recent data presented by Johnson et al. [22] reveal that the closest relatives of ants are tied to soil or mud for nest-making and provisioning (e.g. Ampulicidae, the cockroach wasps; Sphecidae, the digger wasps, mud dauber wasps, and relatives). This conclusion provides support for the hypothesis that ants' origins are in the soil, as it suggests that the habits of larval provisioning and nestmaking are prerequisites for the evolution of sociality in Hymenoptera. These factors were largely neglected in the discussion of ant origins until the recent discovery that ants are more closely related to the predatory wasps that comprise the earliest branching lineages of Apoidea (Bees, sensu lato) than to the ectoparasitoid wasps (which do not construct nests), as previously hypothesized [22]. We can infer that the social lineages of the aculeates modified this lifestyle to actively provision underground burrows larvae in nest sites with paralyzed prey or other resources such as pollen. Several of these lineages evolved into the eusocial Hymenoptera we are familiar with today: (i.e. some wasps, some bees and all ants).

#### Out of the wet, hot, ground?

Arguments for other lineages that arose in the wet tropics suggest subsequent radiation into drier, cooler realms [41]–[43]. Several authors suggest that lower diversity in these latter habitats reflects recent colonizations and more frequent extinction [44]–[45]. A similar tropical origin argument is implicit in the Dynastic Succession model for the rise of ants, and is supported by recent biogeographic analyses [15]. However, our ability to detect the signature of such historic transitions depends on phylogenetic signal strength. Our results show that related ant genera are no more likely than unrelated genera to occupy similar biomes (Table 4). This lack of signal in our dataset prevents reliable reconstruction of the ancestral biome of ants.

**Table 4:** Phylogenetic signal of habitat strata and biome in ants.

Trait	K	P-value
Biome	0.090	0.1458
Strata (6-state)	0.434	0.0001*
Any soil spp.	0.363	0.0001*
Any arboreal spp.	0.153	0.0004*

Phylogenetic signal, presented as Blomberg's K statistic, for habitat traits in ant genera (reconstructed with outgroups). Significance of < 0.05 is designated with an asterisk.

While our data and methods have not offered new insight into the biome in which ants arose, phylogenies are not the only evidence as to the early biomes in which ants lived. The fossil record provides some indication as to the biomes in which early ants have been found, if not necessarily the biome in which the first ants arose. The paleo-reconstructions of the environments in which the earliest ant fossils have been retrieved suggest warm temperate to tropical climates [44]–[45], which were more expansive during the Cretaceous period compared to what is observed today [26]. Nonetheless, the diversity of fossils in these early strata suggests the origin of ants was even earlier [17], [18], [19].

The conclusions from this study contribute to a broader understanding of the early evolution of ants. Looking toward the future, we anticipate that even more insight will be gained by revisiting these questions with a more complete sample of ant genera, as well as inclusion of targeted outgroups that represent the hymenopteran lineages most closely related to ants.

*Conclusions*—Based on recent phylogenetic data we reconstruct a scenario for the ancestral habitat of ants that differs from the dominant paradigm. Rather than finding support for the early rise of ants as surface-dwelling, we find evidence that early ants evolved underground and subsequently transitioned into leaf litter and forest canopies. These findings provide new understanding of the early lifestyle of the most successful eusocial lineage on Earth and invite new questions about their evolutionary trajectory. From an apparently underground origin, perhaps similar to that of their predaceous or parasitic wasp relatives, ants emerged from the ground to diversify and colonize nearly every habitat and biome on Earth.

## Supporting Information

Table S1 **Habitat strata of included ant taxa.** Taxa included in this study are listed in column one by the names used in the original paper (Brady et al. 2006). These represent 151 ant species which correspond to 134 genera. Notes in column two highlight taxa that belong to generic lineages other than those indicated by the name in column one. These notes identify multiple species that belong to the same major lineage, species known to represent different major lineages within a genus (indicated by 1, 2, 3), and those that have undergone nomenclatural changes. Genera are coded in column three according to the habitat in which they are known to occur: soil (A), surface (B), arboreal (C). Column four provides references for generic-level habitat information (full references are listed below table).
(DOCX)

Table S2 **Transition rates for habitat states and biome.** Included are MCMC and ML rate coefficients between all six habitat states, ML rate coefficients between four biome states, and ML rate coefficients for two strata, coded as binary states (subterranean, arboreal). Transitions are listed from the starting to ending state (e.g. 01  =  state 0 -> state 1), with six-state habitat strata coded as 0  =  soil only, 1  =  soil + litter, 2  =  litter only, 3  =  litter + arboreal, 4  =  arboreal only, 5  =  soil + litter + arboreal. Biome is coded as 0  =  tropical + subtropical, 1  =  subtropical, 2  =  subtropical + temperate, 3  =  tropical + subtropical + temperate.
(DOCX)
