# Supplementary material for: Tracing the Rise of Ants - Out of the Ground
Source: PLoS One. 2013 Dec 26;8(12):e84012. doi: 10.1371/journal.pone.0084012 (PMC3873401; doi:10.1371/journal.pone.0084012)
Supplement: Table S1 — Habitat strata of included ant taxa. Taxa included in this study are listed in column one by the names used in the original paper (Brady et al. 2006). These represent 151 ant species which correspond to 134 genera. Notes in column two highlight taxa that belong to generic lineages other than those indicated by the name in column one. These notes identify multiple species that belong to the same major lineage, species known to represent different major lineages within a genus (indicated by 1, 2, 3), and those that have undergone nomenclatural changes. Genera are coded in column three according to the habitat in which they are known to occur: soil (A), surface (B), arboreal (C). Column four provides references for generic-level habitat information (full references are listed below table). (DOCX) [file pone.0084012.s001.docx]

**TABLE S1.** **Habitat strata of included ant taxa.** Taxa included in this study are listed in column one by the names used in the original paper (Brady et al. 2006). These represent 151 ant species which correspond to 134 genera. Notes in column two highlight taxa that belong to generic lineages other than those indicated by the name in column one. These notes identify multiple species that belong to the same major lineage, species known to represent different major lineages within a genus (indicated by 1, 2, 3), and those that have undergone nomenclatural changes. Genera are coded in column three according to the habitat in which they are known to occur: soil (A), surface (B), arboreal (C). Column four provides references for generic-level habitat information (full references are listed below table).

| **Species name (Brady et al. 2006)** | **Taxonomic Notes** | **Habitat Code  (for genus) A: Soil, B: Surface, C: Arboreal** | **Reference(s)** |
| --- | --- | --- | --- |
| *Acanthognathus ocellatus* |  | A | Galvis & Fernández, 2009 |
| *Acanthoponera minor* |  | B | Brown, 2000 |
| *Acanthostichus kirbyi* |  | A | Brown, 2000 |
| *Acromyrmex versicolor* |  | B | Brown, 2000 |
| *Acropyga acutiventris* |  | A | Brown, 2000 |
| *Adetomyrma MAD02* |  | A | PS Ward, Pers. comm. |
| *Aenictogiton ZAM02* |  | A | PS Ward, Pers. comm. |
| *Aenictus ceylonicus* | *Aenictus* | AB | Weeyawat & Yamane, 2011 |
| *Aenictus eugenii* | *Aenictus* | AB | Weeyawat & Yamane, 2011 |
| *Amblyopone mutica* | *Amblyopone 1* | A | Brown, 1960 |
| *Amblyopone pallipes* | *Amblyopone 2* | A | Brown, 1960 |
| *Aneuretus simoni* |  | B | Jayasuriya & Traniello, 1986 |
| *Anochetus madagascarensis* |  | BC | Brown, 2000 |
| *Anonychomyrma gilberti* |  | BC | Brown, 2000 |
| *Anoplolepis gracilipes* |  | B | Brown, 2000 |
| *Aphaenogaster albisetosa* | *Aphaenogaster 1* | B | Brown, 2000 |
| *Aphaenogaster occidentalis* | *Aphaenogaster 2* | B | Brown, 2000 |
| *Aphaenogaster swammerdami* | *Aphaenogaster 3* | B | Brown, 2000 |
| *Apomyrma stygia* |  | A | Brown, 2000 |
| *Apterostigma auriculatum* |  | BC | Brown, 2000 |
| *Azteca ovaticeps* |  | C | Brown, 2000 |
| *Basiceros manni* |  | B | Brown, 2000 |
| *Brachymyrmex depilis* |  | BC | Brown, 2000 |
| *Calomyrmex albertisi* |  | B | Brown, 2000 |
| *Camponotus BCA01* | *Colobopsis* | C | PS Ward, Pers. comm. |
| *Camponotus conithorax* | *Colobopsis* | C | PS Ward, Pers. comm. |
| *Camponotus hyatti* | *Camponotus* | BC | Brown, 2000 |
| *Camponotus maritimus* | *Camponotus* | BC | Brown, 2000 |
| *Cardiocondyla mauritanica* |  | B | Mackay 1995 |
| *Cataulacus MAD02* |  | C | Brown, 2000 |
| *Centromyrmex sellaris* |  | A | Brown, 2000 |
| *Cerapachys augustae* | *Cerapachys 1* | A | Brown, 2000 |
| *Cerapachys larvatus* | *Cerapachys 2* | BC | Brown, 2000 |
| *Cerapachys sexspinus* | *Cerapachys 3* | A | Brown, 2000 |
| *Cheliomyrmex cf morosus* |  | A | Brown, 2000 |
| *Concoctio concenta* |  | A | PS Ward, Pers. Comm. |
| *Crematogaster emeryana* |  | BC | Brown, 2000 |
| *Cylindromyrmex striatus* |  | AB | Brown, 2000 |
| *Daceton armigerum* |  | C | Brown, 2000 |
| *Discothyrea MAD07* |  | A | Brown, 2000 |
| *Dolichoderus scabridus* |  | BC | Brown, 2000 |
| *Dorylus helvolus* | *Dorylus* | AB | Kronauer et al. 2007 |
| *Dorylus laevigatus* | *Dorylus* | AB | Kronauer et al. 2007 |
| *Dorymyrmex bicolor* |  | B | Trager, 1988 |
| *Eciton vagans* |  | AB | Brown, 2000 |
| *Ectatomma opaciventre* |  | B | Brown, 2000 |
| *Eurhopalothrix bolaui* |  | A | Longino, 2013 |
| *Eutetramorium mocquerysi* |  | B | Brown, 2000 |
| *Forelius pruinosus* |  | B | Brown, 2000 |
| *Formica moki* |  | B | Brown, 2000 |
| *Gnamptogenys striatula* |  | AB | Brown, 2000 |
| *Heteroponera panamensis* |  | A | Brown, 2000 |
| *Hypoponera opacior* | *Hypoponera* | A | Taylor, 1967 |
| *Hypoponera sakalava* | *Hypoponera* | A | Taylor, 1967 |
| *Lasius californicus* |  | AB | Brown, 2000 |
| *Leptanilla GRE01* | *Leptanilla* | A | Ward & Sumnicht 2012 |
| *Leptanilla RSA01* | *Leptanilla* | A | Ward & Sumnicht 2012 |
| *Leptanilloides mckennae* | *Leptanilloides* | A | Brown, 2000 |
| *Leptanilloides nomada* | *Leptanilloides* | A | Brown, 2000 |
| *Leptogenys diminuta* |  | AB | J. Lattke, Pers. com |
| *Leptomyrmex AUS01* | *Leptomyrmex* | B | Brown, 2000 |
| *Leptomyrmex erythrocephalus* | *Leptomyrmex* | B | Brown, 2000 |
| *Leptothorax muscorum complex* |  | B | Brown, 2000 |
| *Linepithema humile* |  | B | Brown, 2000 |
| *Liometopum apiculatum* | *Liometopum* | BC | Brown, 2000 |
| *Liometopum occidentale* | *Liometopum* | BC | Brown, 2000 |
| *Loboponera politula* |  | A | PS Ward, Pers. Comm. |
| *Manica bradleyi* |  | B | Brown, 2000 |
| *Mayriella ebbei* |  | AB | Wheeler, 1935 |
| *Meranoplus cf radamae* |  | B | Brown, 2000 |
| *Messor andrei* | *Messor 1* | B | Brown, 2000 |
| *Messor denticornis* | *Messor 2* | B | Brown, 2000 |
| *Metapone madagascarica* |  | A | Brown, 2000 |
| *Microdaceton tibialis* |  | A | PS Ward, Pers. Comm. |
| *Monomorium ergatogyna* |  | ABC | Brown, 2000 |
| *Myrcidris epicharis* |  | C | Brown, 2000 |
| *Myrmecia pyriformis* |  | B | Brown, 2000 |
| *Myrmecina graminicola* |  | AB | Shattuck, 1990 |
| *Myrmecocystus flaviceps* |  | B | Brown, 2000 |
| *Myrmelachista JTL01* |  | C | Brown, 2000 |
| *Myrmica striolagaster* | *Myrmica* | B | Brown, 2000 |
| *Myrmica tahoensis* | *Myrmica* | B | Brown, 2000 |
| *Myrmicaria exigua* |  | BC | Brown, 2000 |
| *Myrmicocrypta cf infuscata* |  | B | Sosa-Calvo & Schultz, 2010 |
| *Myrmoteras iriodum* |  | B | Brown, 2000 |
| *Mystrium mysticum* |  | A | Brown, 2000 |
| *Neivamyrmex nigrescens* |  | AB | Brown, 2000 |
| *Nesomyrmex echinatinodis* |  | C | Bolton, 2003 |
| *Nothomyrmecia macrops* |  | B | Brown, 2000 |
| *Notoncus capitatus* |  | B | Brown, 2000 |
| *Notostigma carazzii* |  | B | Brown, 2000 |
| *Odontomachus coquereli* |  | B | Brown, 2000 |
| *Odontoponera transversa* |  | B | Brown, 2000 |
| *Oecophylla smaragdina* |  | C | Brown, 2000 |
| *Onychomyrmex hedleyi* |  | B | Wheeler, 1916; Miyata et al. 2009 |
| *Opisthopsis respiciens* |  | BC | Brown, 2000 |
| *Orectognathus versicolor* |  | BC | Bolton, 1999 |
| *Pachycondyla sikorae* |  | B | Brown, 2000 |
| *Papyrius nitidus* |  | B | Brown, 2000 |
| *Paraponera clavata* |  | B | Brown, 2000 |
| *Paratrechina hystrix* | *Nylanderia* | B | Brown, 2000 |
| *Pheidole clydei* | *Pheidole* | B | Brown, 2000 |
| *Pheidole hyatti* | *Pheidole* | B | Brown, 2000 |
| *Pheidologeton affinis* |  | B | Brown, 2000 |
| *Philidris cordatus* |  | C | Brown, 2000 |
| *Pilotrochus besmerus* |  | A | Bolton, 1984. |
| *Platythyrea mocquerysi* | *Platythyrea* | BC | Brown, 2000 |
| *Platythyrea punctata* | *Platythyrea* | BC | Brown, 2000 |
| *Plectroctena ugandensis* |  | A | Brown, 2000 |
| *Pogonomyrmex subdentatus* |  | B | Brown, 2000 |
| *Polyergus breviceps* |  | B | Brown, 2000 |
| *Polyrhachis Cyrto01* | *Polyrachis* | BC | PS Ward, Pers. Comm. |
| *Polyrhachis Hagio01* | *Polyrachis* | BC | Kohout, 2000 |
| *Prenolepis albimaculata* | *Prenolepis 1* | B | Brown, 2000 |
| *Prenolepis imparis* | *Prenolepis 2* | B | Brown, 2000 |
| *Prionopelta MAD01* |  | A | Brown, 2000 |
| *Probolomyrmex tani* |  | A | Brown, 2000 |
| *Proceratium MAD08* | *Proceratium* | A | Brown, 1980 |
| *Proceratium stictum* | *Proceratium* | A | Brown, 1980 |
| *Procryptocerus scabriusculus* |  | C | Brown, 2000 |
| *Protanilla JAP01* |  | A | Borowiec,  2011 |
| *Psalidomyrmex procerus* |  | A | Brown, 2000 |
| *Pseudolasius australis* |  | AB | LaPolla, 2010 |
| *Pseudomyrmex gracilis* |  | C | Brown, 2000 |
| *Pyramica hoplites* |  | AB | Baroni Urbani & De Andrade, 2007 |
| *Rhopalomastix rothneyi* |  | C | Brown, 2000 |
| *Rhytidoponera chalybaea* |  | B | Brown, 2000 |
| *Simopelta cf pergandei* |  | AB | Brown, 2000 |
| *Simopone marleyi* |  | C | Bolton & Fisher, 2012 |
| *Solenopsis molesta* | *Solenopsis* | ABC | Brown, 2000 |
| *Solenopsis xyloni* | *Solenopsis* | ABC | Brown, 2000 |
| *Sphinctomyrmex steinheili* |  | A | Shattuck, 1999 |
| *Stenamma dyscheres* |  | AB | Branstetter, 2013 |
| *Strumigenys dicomas* |  | ABC | Brown, 2000 |
| *Tapinoma sessile* |  | BC | Brown, 2000 |
| *Tatuidris ECU01* |  | A | Donoso, 2012 |
| *Technomyrmex difficilis* |  | BC | Brown, 2000 |
| *Technomyrmex MAD05* | *Technomyrmex* | BC | Brown, 2000 |
| *Temnothorax rugatulus* |  | BC | PS Ward, Pers. Comm. |
| *Terataner MAD02* |  | C | Bolton, 1981 |
| *Tetramorium caespitum* | *Tetramorium* | BC | Brown, 2000 |
| *Tetramorium validiusculum* | *Tetramorium* | BC | Brown, 2000 |
| *Tetraponera punctulata* | *Tetraponera 1* | C | Brown, 2000 |
| *Tetraponera rufonigra* | *Tetraponera 2* | C | Brown, 2000 |
| *Thaumatomyrmex atrox* |  | A | Brown, 2000 |
| *Trachymyrmex arizonensis* |  | B | Brown, 2000 |
| *Turneria bidentata* |  | C | Brown, 2000 |
| *Typhlomyrmex rogenhoferi* |  | A | Brown, 2000 |
| *Vollenhovia emeryi* |  | B | Brown, 2000 |
| *Wasmannia auropunctata* |  | BC | Brown, 2000 |
| *Xenomyrmex floridanus* |  | C | Brown, 2000 |

**References (Supplementary Table A):**

1. Baroni Urbani C, De Andrade ML (2007). The ant tribe Dacetini: limits and constituent genera, with descriptions of new species (Hymenoptera, Formicidae). *Annali del Museo Civico di Storia Naturale "Giacomo Doria"* 99: 1-191.
2. Bolton B (2003) Synopsis and classification of Formicidae. *Mem Amer Entomol Inst* 71:1-370.
3. Bolton B (1999) Ant genera of the tribe Dacetonini (Hymenoptera: Formicidae). *J Nat Hist* 33:1639-1689.
4. Bolton B (1984) Diagnosis and relationships of the myrmicine ant genus *Ishakidris* gen. n. (Hymenoptera: Formicidae). *Syst Entomol* 9: 373-382.
5. Bolton B (1981) A revision of six minor genera of Myrmicinae (Hymenoptera: Formicidae) in the Ethiopian zoogeographical region. *Bull British Mus (Natural History). Entomology.* 43: 245-307.
6. Bolton B, Fisher BL (2012) Taxonomy of the cerapachyine ant genera *Simopone* Forel, *Vicinopone* gen. n. and *Tanipone* gen. n. (Hymenoptera: Formicidae). *Zootaxa* 3283: 1–101.
7. Borowiec ML, Schulz A, Alpert GD, Banar P (2011) Discovery of the worker caste and descriptions of two new species of *Anomalomyrma* (Hymenoptera: Formicidae: Leptanillinae) with unique abdominal morphology. *Zootaxa* 2810: 1-14.
8. Branstetter MG (2013) Revision of the Middle American clade of the ant genus *Stenamma* Westwood (Hymenoptera, Formicidae, Myrmicinae). *ZooKeys* 295:1-277.
9. Brown WL (2000) Diversity of ants. Pp. 45-79 in: Agosti D, Majer JD, Alonso LE, Schultz TR (eds.) 2000. Ants. Standard methods for measuring and monitoring biodiversity. Washington: Smithsonian Institution Press, 280 pp.
10. Brown WL (1980) A remarkable new species of *Proceratium*, with dietary and other notes on the genus (Hymenoptera: Formicidae). *Psyche* 86: 337-346.
11. Brown WL (1960) Contributions towards a reclassification of the Formicidae. III. Tribe Amblyoponini (Hymenoptera). *Bull Mus Comp Zool* *Harvard* 122: 145-230.
12. Donoso DA (2012) Additions to the taxonomy of the armadillo ants (Hymenoptera, Formicidae, *Tatuidris*). *Zootaxa* 3503: 61–81.
13. Galvis JP, Fernández F (2009) Ants of Colombia X. *Acanthognathus* with the description of a new species (Hymenoptera: Formicidae). *Rev Colombiana Entomol* 35:245-249.
14. Jaitrong W, Yamane S (2011) Synopsis of *Aenictus* species groups and revision of the *A. currax* and *A. laeviceps* groups in the eastern Oriental, Indo-Australian, and Australasian regions. (Hymenoptera: Formicidae: Aenictinae). *Zootaxa* 3128: 1-46.
15. Jayasuriya AK, Traniello JFA (1986) The biology of the primitive ant *Aneuretus simoni* (Emery) (Formicidae: Aneuretinae). I. Distribution, abundance, colony structure, and foraging ecology. *Insectes Sociaux* 32: 363-374.
16. Kohout RJ (2000) A review of the distribution of the *Polyrachis* and *Echinopla* ants of the Queensland wet tropics (Hymenoptera: Formicidae: Formicinae). *Mem QLD Mus* 46: 183-209.
17. Kronauer D, Schöning C, Vilhelmsen LB, Boomsma JJ (2007) A molecular phylogeny of *Dorylus* army ants provides evidence for multiple evolutionary transitions in foraging niche. *BMC Evol Biol*. 7: 56-66.
18. LaPolla JS, Brady SG, Shattuck, SO (2010) Phylogeny and taxonomy of the *Prenolepis* genus-group of ants (Hymenoptera: Formicidae). *Syst Entomol* 35: 118-131.
19. Longino JT (2013) A review of the Central American and Caribbean Species of the ant genus *Eurhopalothrix* Brown and Kempf, 1961 (Hymenoptera, Formicidae), with a key to New World species. *Zootaxa* 3693:101-151.
20. Mackay WP (1995) New distributional records for the ant genus *Cardiocondyla* in the new world (Hymenoptera: Formicidae). *Pan-Pacific Entomologist* 71:169-172*.*
21. Miyata H, Hirata M, Azuma N, Murakami T, Higashi S (2009) Army ant behaviour in the poneromorph hunting ant *Onychomyrmex hedleyi* Emery (Hymenoptera: Formicidae; Amblyoponinae). *Aust J Entomol* 48: 47-52.
22. Shattuck SO (1999) Australian ants. Their biology and identification. Collingwood, Victoria: CSIRO Publishing, xi + 226 pp. p 62-63.
23. Shattuck SO (1990) A revision the Australian species of the ant genus *Myrmecina* (Hymenoptera: Formicidae). *Zootaxa* 2146: 1-21.
24. Sosa-Calvo J, Schultz TR (2010) Three remarkable new fungus-growing ant species of the genus *Myrmicocrypta* (Hymenoptera: Formicidae), with a reassessment of the characters that deﬁne the genus and its position within the Attini. *Ann Entomol Soc America* 103: 181-195.
25. Taylor RW (1967) A monographic revision of the ant genus *Ponera* Latreille (Hymenoptera: Formicidae). *Pacific Insects Monograph* 13: 1-112.
26. Trager JC (1988) A Revision of *Conomyrma* (Hymenoptera: Formicidae) from the Southeastern United States, Especially Florida, with Keys to the Species. *FL Entomol* 71: 11-29.
27. Ward PS, Sumnicht TP (2012) Molecular and morphological evidence for three sympatric species of *Leptanilla* on the Greek island of Rhodes. *Myrmecol News* 17:5-11.
28. Wheeler WM (1935) The Australian ant genus *Mayriella* Forel. *Psyche* 42: 151-160.
29. Wheeler WM (1916) The Australian ants of the genus *Onychomyrmex*. *Bull Mus Comp Zool* *Harvard* 60:45-54.
