# Supplementary material for: Tracing the Rise of Ants - Out of the Ground
Source: PLoS One. 2013 Dec 26;8(12):e84012. doi: 10.1371/journal.pone.0084012 (PMC3873401; doi:10.1371/journal.pone.0084012)
Supplement: Table S2 — Transition rates for habitat states and biome. Included are MCMC and ML rate coefficients between all six habitat states, ML rate coefficients between four biome states, and ML rate coefficients for two strata, coded as binary states (subterranean, arboreal). Transitions are listed from the starting to ending state (e.g. 01 = state 0 -> state 1), with six-state habitat strata coded as 0 = soil only, 1 = soil + litter, 2 = litter only, 3 = litter + arboreal, 4 = arboreal only, 5 = soil + litter + arboreal. Biome is coded as 0 = tropical + subtropical, 1 = subtropical, 2 = subtropical + temperate, 3 = tropical + subtropical + temperate. (DOCX) [file pone.0084012.s002.docx]

**TABLE S2**. **Transition rates for habitat states and biome**. Included are MCMC and ML rate coefficients between all six habitat states, ML rate coefficients between four biome states, and ML rate coefficients for two strata, coded as binary states (subterranean, arboreal). Transitions are listed from the starting to ending state (e.g. 01 = state 0 -> state 1), with six-state habitat strata coded as 0 = soil only, 1 = soil + litter, 2 = litter only, 3 = litter + arboreal, 4 = arboreal only, 5 = soil + litter + arboreal. Biome is coded as 0 = tropical + subtropical, 1 = subtropical, 2 = subtropical + temperate, 3 = tropical + subtropical + temperate.

|  | **Strata (6-state)** | | **Biome (4-state)** | |  | **Subterranean (2-state)** | **Arboreal (2-state)** |
| --- | --- | --- | --- | --- | --- | --- | --- |
| **Transition** | MCMC | ML | **Transition** | ML | **Transition** | ML | ML |
| **01** | 0.000845 | 0.086089 | **01** | 0.000649 | **No to yes** | 0.00179596 | 0.00147210 |
| **02** | 0.000961 | 0.001400 | **02** | 0.001243 | **Yes to No** | 0.00085215 | 0.00493486 |
| **03** | 0.000125 | 0.000035 | **03** | 0.004774 |  |  |  |
| **04** | 0.000143 | 0.000495 | **10** | 0.048017 |  |  |  |
| **05** | 0.000161 | 0.002009 | **12** | 0.170519 |  |  |  |
| **10** | 0.002626 | 0.194157 | **13** | 0.550474 |  |  |  |
| **12** | 0.826582 | 1.360031 | **20** | 0.226844 |  |  |  |
| **13** | 0.766279 | 0.874630 | **21** | 0.506303 |  |  |  |
| **14** | 0.856877 | 0.838966 | **23** | 1.057840 |  |  |  |
| **15** | 0.430456 | 0.365113 | **30** | 0.001291 |  |  |  |
| **20** | 0.000841 | 0.000744 | **31** | 0.017154 |  |  |  |
| **21** | 0.207821 | 0.329771 | **32** | 0.011621 |  |  |  |
| **23** | 0.312928 | 0.416458 |  |  |  |  |  |
| **24** | 0.426664 | 0.462031 |  |  |  |  |  |
| **25** | 0.025419 | 0.119841 |  |  |  |  |  |
| **30** | 0.000845 | 0.001824 |  |  |  |  |  |
| **31** | 0.744330 | 0.446609 |  |  |  |  |  |
| **32** | 0.571454 | 0.882126 |  |  |  |  |  |
| **34** | 0.635370 | 0.926757 |  |  |  |  |  |
| **35** | 0.102579 | 0.313720 |  |  |  |  |  |
| **40** | 0.000795 | 0.004920 |  |  |  |  |  |
| **41** | 0.555185 | 0.456070 |  |  |  |  |  |
| **42** | 0.761586 | 0.984444 |  |  |  |  |  |
| **43** | 0.936644 | 1.245587 |  |  |  |  |  |
| **45** | 0.373945 | 0.329682 |  |  |  |  |  |
| **50** | 0.001556 | 0.171391 |  |  |  |  |  |
| **51** | 0.739573 | 2.078355 |  |  |  |  |  |
| **52** | 0.943967 | 2.013434 |  |  |  |  |  |
| **53** | 0.663791 | 1.758321 |  |  |  |  |  |
| **54** | 0.639667 | 1.820429 |  |  |  |  |  |
